# Diffusion Tensor Imaging Reveals Microstructural Heterogeneity of Normal-Appearing White Matter and Related Cognitive Dysfunction in Glioma Patients

**DOI:** 10.3389/fonc.2019.00536

**Published:** 2019-06-26

**Authors:** Kerstin Jütten, Verena Mainz, Siegfried Gauggel, Harshal Jayeshkumar Patel, Ferdinand Binkofski, Martin Wiesmann, Hans Clusmann, Chuh-Hyoun Na

**Affiliations:** ^1^Department of Neurosurgery, RWTH Aachen University, Aachen, Germany; ^2^Faculty of Medicine, Institute of Medical Psychology and Medical Sociology, RWTH Aachen University, Aachen, Germany; ^3^Division of Clinical Cognitive Sciences, RWTH Aachen University, Aachen, Germany; ^4^Research Center Jülich GmbH, Institute of Neuroscience and Medicine (INM-4), Jülich, Germany; ^5^Jülich Aachen Research Alliance, Translational Brain Medicine, Aachen, Germany; ^6^Department of Diagnostic and Interventional Neuroradiology, RWTH Aachen University, Aachen, Germany

**Keywords:** glioma, diffusion tensor imaging, normal-appearing white matter, microstructural integrity, neuropsychology, IDH mutation

## Abstract

Immunohistochemical data based on isocitrate–dehydrogenase (IDH) mutation status have redefined glioma as a whole-brain disease, while occult tumor cell invasion along white matter fibers is inapparent in conventional magnetic resonance imaging (MRI). The functional and prognostic impact of focal glioma may however relate to the extent of white matter involvement. We used diffusion tensor imaging (DTI) to investigate microstructural characteristics of whole-brain normal-appearing white matter (NAWM) in relation to cognitive functions as potential surrogates for occult white matter involvement in glioma. Twenty patients (12 IDH-mutated) and 20 individually matched controls were preoperatively examined using DTI combined with a standardized neuropsychological examination. Tumor lesions including perifocal edema were masked, and fractional anisotropy (FA) as well as mean, radial, and axial diffusivity (MD, RD, and AD, respectively) of the remaining whole-brain NAWM were determined by using Tract-Based Spatial Statistics and histogram analyses. The relationship between extratumoral white matter integrity and cognitive performance was examined using partial correlation analyses controlling for age, education, and lesion volumes. In patients, mean FA and AD were decreased as compared to controls, which agrees with the notion of microstructural impairment of NAWM in glioma patients. Patients performed worse in all cognitive domains tested, and higher anisotropy and lower MD and RD values of NAWM were associated with better cognitive performance. In additional analyses, IDH-mutated and IDH-wildtype patients were compared. Patients with IDH-mutation showed higher FA, but lower MD, AD, and RD values as compared to IDH-wildtype patients, suggesting a better preserved microstructural integrity of NAWM, which may relate to a less infiltrative nature of IDH-mutated gliomas. Diffusion-based phenotyping and monitoring microstructural integrity of extratumoral whole-brain NAWM may aid in estimating occult white matter involvement and should be considered as a complementary biomarker in glioma.

## Introduction

While the extent of tumor resection is regarded as an important therapy-dependent prognostic factor, the infiltrative nature of diffuse glioma remains a diagnostic and therapeutic problem and an obstacle to curative treatment. Infiltrative glioma growth preventing the definition of clear tumor borders has been described early on based on histological findings (1). With identification of the isocitrate–dehydrogenase (IDH) mutation in codon 132 of IDH1 (or infrequently in codon 172 of IDH2) as being the most common molecular genetic alteration in grade II and grade III gliomas, immunohistochemical data using antibodies specific to IDH1 R132H mutant protein have revealed a more widespread tumor cell distribution than expected. Occult tumor cell invasion has thereby been delineated even in areas remote to the primary tumor site, which had macro- and microscopically appeared unaffected (2). These findings have redefined cerebral glioma as a systemic rather than focal brain disease, which is supported by cellular observations of diffusively invading tumor cells expanding to extensive intracerebral networks (3).

In view of these findings, standard treatment and disease monitoring strategies focusing only on the focal tumor and its borders seem to be insufficient. With immunohistochemical analyses being limited to post-mortem IDH-mutant glioma, and conventional magnetic resonance imaging (MRI) incapable of delineating diffuse tumor cell migration, the extent of occult tumor cell burden has not been determined *in vivo*, although it might offer prognostically valuable information. As diffuse infiltrative glioma growth has histologically been characterized to occur typically along white matter (WM) tracts (4), diffusion tensor imaging (DTI) appears of particular interest. It provides indirect information about the microstructural WM architecture and its integrity *in vivo*, based on water diffusion properties in the intra- and extracellular space (5). This notion is based on a simplified model, in which water diffusion within a given voxel can be described as diffusion tensor or ellipsoid. The shape of the ellipsoid is characterized by three eigenvalues (λ1–3), providing measures of the three primary axes of the ellipsoid. Diffusivity along the principal axis (λ1) is called axial diffusivity (AD), and diffusivity along the two minor axes (λ2 and 3) is called radial diffusivity (RD) (6, 7). While AD has been related to axonal integrity, RD has been assumed to provide information about the degree of myelination (8, 9). These eigenvalues can be used to calculate scalar DTI summary measures such as fractional anisotropy (FA), reflecting the orientation preference of water diffusion within a given voxel (5, 10), and mean diffusivity (MD), reflecting the mean amount of water diffusion irrespective of its direction (11). While higher FA and AD in WM have been linked to preserved fiber integrity, increases in MD and RD have been related to structural disintegration (12).

Studies on glioma-induced WM changes in regions remote to the primary tumor site are only sparse, but suggest DTI to allow tracing glioma cell infiltration into brain regions distant to the primary tumor site, which is inapparent in conventional imaging (13–16). Previous studies in this regard were based on region-of-interest (ROI) analyses, in which DTI metrics were determined locally at assumed tumor projections along predefined WM structures such as the corticospinal tract or in peritumoral areas, with restriction to observer-dependent predefined regions. Based on peritumoral anisotropy and diffusivity measures (17), the more favorable prognosis in IDH-mutated glioma (18, 19) has been related to a less invasive behavior as compared to IDH-wildtype gliomas. A ROI-independent diffusion-based microstructural characterization of whole-brain normal-appearing white matter (NAWM) under consideration of IDH-mutation status and in relation to cognitive functions has, to the best of our knowledge, not yet been attempted.

While glioma patients with “non-eloquent” tumor locations do not exhibit apparent deficits in basic sensory-motor functions, cognitive impairment has been described across different tumor locations in most of the patients already at the time of diagnosis (20, 21), which has been linked to early WM involvement (22). While patients with lower-grade gliomas initially may present with impressive tumor volumes but only minor cognitive dysfunctions, patients with high-grade tumors at comparable anatomical sites can be more severely impaired (21, 23). This might be based on different dynamics of tumor-induced cortical deafferentation or reorganization processes, depending on the local tumor growth rate. Alternatively, this could also be related to varying degrees of occult systemic tumor cell burden, with immunohistochemically proven diffuse tumor cell migration into brain regions remote to the primary tumor site (2).

This study aimed to preoperatively investigate microstructural and functional characteristics of whole brain NAWM as potential measures of occult systemic WM involvement in glioma. In order to restrict analyses to NAWM, contrast-enhancing and non-contrast-enhancing tumor lesions including any perifocal T2- or T1-weighted signal alterations such as perifocal edema were captured in tumor lesion masks and excluded from analyses. We hypothesized that global microstructural WM integrity and related cognitive functions would be compromised in glioma. Moreover, WM integrity was assumed to be more preserved in IDH-mutated than in IDH-wildtype glioma, which would agree with the notion of a more favorable prognosis and less infiltrative nature of IDH-mutated glioma.

## Materials and Methods

### Subjects

Twenty patients (mean age: 44.8 years, *SD* = 15.5, 13 males, 12 IDH-mutated) and 20 matched healthy controls (mean age: 45.3 years, *SD* = 15.9, 13 males) were prospectively enrolled in the study at a single university hospital center. All subjects underwent anatomical MRI, DTI, and standardized neuropsychological testing. Patients were examined preoperatively and histopathological diagnoses were determined (based on tumor specimens obtained by biopsy or tumor resection) according to the revised WHO tumor classification of 2016 (24), integrating imaging, histological, and molecular genetic criteria. IDH-mutation status was determined by immunohistochemical analysis with identification of the IDH1 R132H mutation or, when results were negative or unequivocal, by additional DNA analysis using next-generation sequencing. IDH-mutation status was defined by presence of mutations of IDH1 on exon 4, codon 132 or of IDH2 on exon 4, codon 172.

All participants gave written informed consent prior to study enrollment. This study was approved (EK 294/15) by the local ethics committee, and conducted in accordance with the standards of Good Clinical Practice and the Declaration of Helsinki. Only patients >18 and <80 years of age with unilateral supratentorial tumor and a Karnofsky index of >70 were included. All patients except one were naive to tumor-specific treatment prior to enrollment in the study. One patient with presumed low-grade glioma (according to neuroradiological criteria) consented in participation in the study, but finally refrained from surgery, so that histopathological confirmation could not be obtained in this subject. For detailed information on patients' demographics and tumor characteristics (see Table 1).

**Table 1:** Clinical description of included patients.

**Patients**	**IDH-mutation**	**Diagnosis**	**Grade**	**Location**	**Side**	**Volume (in cm^3^)**	**AE**	**Steroids**	**Age (years)**	Education^*^ (years)
1	y	Astrocytoma	II	Temporal	r	30	y	n	30–35	13
2	y	Astrocytoma	II	Frontal	l	51	y	n	20–25	16
3	y	Astrocytoma	II	Parietal	l	64	y	n	55–60	18
4	y	Astrocytoma	II	Frontal	l	158	y	n	26–30	13
5	y	Oligodendro-glioma	II	Frontal	r	2	n	n	36–40	13
6	y	Oligodendro-glioma	II	Frontal	l	22	n	n	26–30	13
7	y	Anaplastic astrocytoma	III	Frontal	l	21	y	y	50–55	13
8	y	Anaplastic astrocytoma	III	Parietal	l	119	y	n	20–25	13
9	y	Anaplastic astrocytoma	III	Frontal	r	155	n	n	30–35	15
10	y	Anaplastic astrocytoma	III	Frontal, insular	r	175	y	y	30–35	13
11	y	Anaplastic oligodendro-glioma	III	Frontal	l	39	y	n	50–55	15
12	y	Anaplastic oligodendro-glioma	III	Frontal	r	96	n	n	30–35	18
13	n	Dysembryo-plastic neuroepithelial tumor	I	Hippocampal	l	24	y	n	40–45	15
14	n	Anaplastic astrocytoma	III	Temporo-parieto-occipital	l	144	n	n	66–70	12
15	n	Glioblastoma multiforme	IV	Fronto-temporal, insular	l	204	y	y	60–65	15
16	n	Glioblastoma multiforme	IV	Temporal, insular	l	111	y	n	56–60	10
17	n	Glioblastoma multiforme	IV	Fronto-temporal, insular	l	145	n	y	66–70	9
18	n	Glioblastoma multiforme	IV	Frontal	r	182	n	y	50–55	12
19	n	Glioblastoma multiforme^a^	IV	Parietal, thalamic	l	65	y	y	50–55	13
20	–	Presumed low-grade glioma^b^	–	Occipital	r	1	n	n	60–65	12

*IDH, isocitrate–dehydrogenase; y, yes; n, no; l, left; r, right; AE, anti-epileptics; m, male; f, female*.

a *Recurrent glioblastoma 5 months after first tumor resection and adjuvant radiochemotherapy*.

b *Patient refrained from surgery so that no histopathological confirmation could be obtained*.

* *Years of education were computed by the sum of years spent for school career and further training/study*.

### Neuropsychological Assessment

All participants underwent a cognitive assessment using a standardized neuropsychological examination. Duration of testing was about 60 min and included the following tests: The Verbal Learning and Memory Test (25) (VLMT) is designed as a list-learning paradigm and included eight trials: five trials (Trials 1–5) of repeated presentation and immediate recall of a list of 15 words (Trial A), followed by the presentation and recall of a second “interference” list of 15 words (Trial B), another trial assessing immediate post-interference recall of list A (Trial 6), and an additional recall trial after a 20-min delay (Trial 7). A final recognition trial (Trial 8) included those words from Lists A and B, and 20 phonologically or semantically similar words to Lists A and B. Participants needed to identify and recognize the words that were part of List A. This study focused on VLMT scores including total learning (the sum of scores for Trials 1–5, VLMT_rec), consolidation performance as number of words forgotten over time (Trial 5 score – Trial 7 score, VLMT_con), and recognition (Trial 8 score, VLMT_recog).

The Attention Network Test (ANT), a modified Posner task (26), is a selective reaction time task that is used to examine three attentional systems, including tonic and phasic alertness, spatial, and executive attention. The participants' task is to react as quickly as possible to directional stimuli (arrows) that are imbedded in special cues or distractors intending to stimulate the various attention components. Specifically, an arrow, which can point to the right or left, appears in the middle of the screen below or above a fixation cross, and participants should indicate that arrow's direction by button press. By varying cues and distracters, responses address either the alertness or spatial (orientation) or executive (conflict) condition. The ANT consists of four blocks and 288 trials, including one practice block (24 trials) as well as three test blocks (96 trials each), which takes participants about 20 min to finish. Specifically, this study focused on overall alertness by investigating mean reaction times (RTs) in seconds (RT of correct trials, ANT_RTcor) as well as the number of errors in the according subtrials of the test (number of errors, ANT_err).

As a test for executive functions, the Trail-Making Test (27) (TMT) was carried out in which participants first have to connect numbers in ascending order (TMT-A). In the second part, numbers and letters are connected alternating in ascending order (TMT-B). RTs in seconds are recorded separately for each part of the test and the difference between the two is regarded to reflect task-switching (difference in RT between TMT-A and TMT-B, TMT_RTexe).

### MRI Data Acquisition

All participants underwent MRI examination on a 3-Tesla Siemens Prisma MRI scanner equipped with a standard 32-channel head coil. The scanning protocol included a 3D T1-weighted as well as a 3D inversion recovery scan for tumor volume segmentation, as well as diffusion-weighted imaging (DWI) to investigate WM integrity. Pulse sequences were as follows: First, a sagittal 3D T1 magnetization-prepared rapid acquisition gradient echo (MPRAGE) sequence was acquired [repetition time (TR) = 2,300 ms, echo time (TE) = 2.01 ms, 176 slices with a slice thickness of 1 mm, flip angle = 9°, field of view (FoV) = 256 mm, voxel size = 1 mm isotropic, and 256 × 256 matrix]. In addition, DWI data were acquired using an echo planar imaging (EPI) sequence (64 diffusion directions with *b*-value = 1,000 s/mm^2^, one b0 image, TR = 6,300 ms; TE = 81 ms; 55 axial slices with 2.4 mm slice thickness, FoV = 216 mm, voxel resolution = 2.4 mm isotropic) and a fluid attenuation inversion recovery (FLAIR) sequence was applied (TR = 4,800 ms, TE = 304.0 ms, number of slices = 160 with 1 mm slice thickness, FoV m s250 mm, and 1 mm isotropic voxel resolution). For tumor identification purposes, a contrast-enhanced, T1-weighted turbo inversion recovery magnitude (TIRM) dark-fluid sequence was acquired (TR = 2,200 ms, TE = 32 ms, slice thickness = 3 mm, flip angle = 150°, FOV = 230 mm, voxel size = 0.9 × 0.9 × 3.0 mm3, matrix = 256 × 256) as well as a T2-weighted TIRM dark-fluid scan (TR = 9,000 ms, TE = 79 ms, slice thickness = 3 mm, flip angle = 150°, FOV = 230 mm, voxel size = 0.9 × 0.9 × 3 mm3, matrix = 256 × 256).

### MRI Data Processing

#### Tumor Masking

In a first step, tumor lesions were segmented semi-automatically on the basis of each patient's anatomical 3D T1-weighted and 3D-FLAIR weighted images using ITK-SNAP software (28). In a second step, tumor lesion masks were manually corrected and lesion volumes (Tvol in cm^3^) were computed. Any apparent tumor lesions including contrast-enhancing and non-contrast-enhancing tumor, as well as any perifocal T2 hyper- or T1 hypointensities including perifocal edema, were included in the lesion masks and excluded from analysis, thereby restricting the analyses to the remaining whole-brain NAWM. In addition, lesion masks were binarized for later use in the normalization procedure of image preprocessing.

#### DTI Preprocessing

The first part of DTI data preprocessing was performed using the FDT diffusion toolbox as implemented in FSL (29) and included distortion and motion correction (eddy current correction) as well as skull stripping (BET brain extraction tool). Diffusion tensors were estimated using DTIFIT, and individual FA, MD, RD, and AD images were created for every subject. Further preprocessing was carried out using SPM12 (30, 31) as implemented in Matlab 9.3. First, subjects' T1-weighted anatomical images were segmented using the segmentation approach, which allows the inclusion of the individual tumor masks for patients as masking image, saving deformation field matrices for standard space as well as subject space (inverse) transformation. After this, diffusion images were co-registered to the corresponding T1-weighted anatomical images. For control subjects, diffusion images were then normalized to their matched patients' diffusion space by means of inverse transformation using the unified segmentation approach (32). Finally, the individual binary tumor mask was applied to patients' FA, MD, RD, and AD images in order to mask out tumor tissue for further analyses. For controls, the tumor mask of the corresponding patient match was applied accordingly, thereby controlling for known WM heterogeneities depending on anatomical site, gender, and age.

#### DTI Metrics

**Figure 1:**
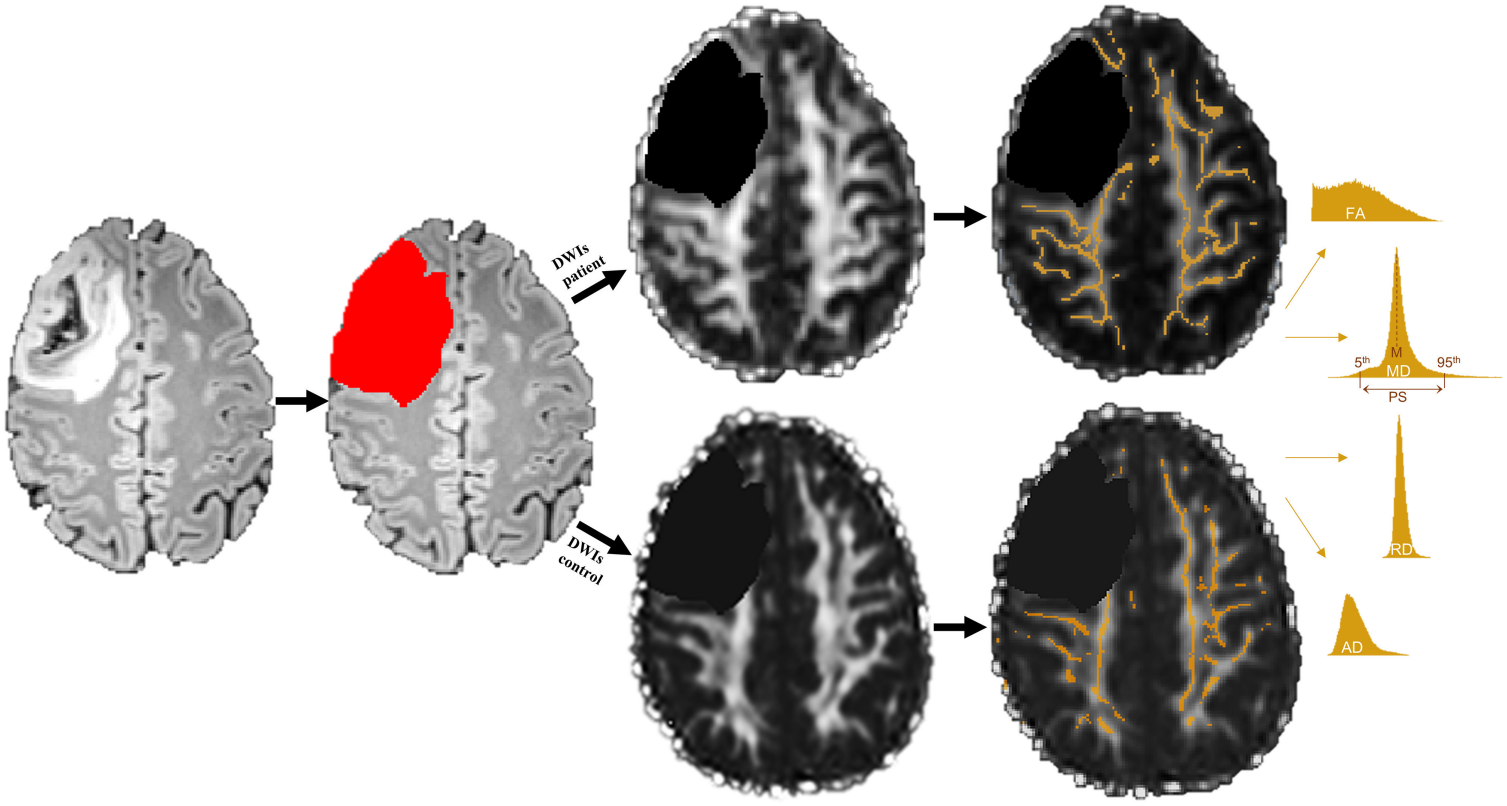
Example of a grade III tumor segmentation based on a fluid attenuation inversion recovery (FLAIR) image. Segmented tumor tissue (red) was excluded in both patients' diffusion weighted images (DWIs) as well as their matched controls' DWIs (black) prior to white matter (WM) skeleton extraction. Only those WM voxels revealing a fractional anisotropy (FA) value of 0.2 or higher were used to create a WM skeleton mask (orange), which was then applied to mean, radial, and axial diffusivity (MD, RD, and AD) maps. Mean (M) and peak width difference between the 95th and 5th percentile (PS) of each skeletonized diffusion parameter were computed by means of histogram analyses.

For between-group comparisons of patients and controls, an approach analogous to TBSS as implemented in FSL was applied. For this purpose, individual diffusion space FA images were skeletonized and thresholded at an FA value of 0.2. The FA-derived skeleton was then applied to MD, RD, and AD diffusion images and masked with patients' WM masks. In order to obtain a most complex characterization of diffusion properties of NAWM, two categories of diffusion values were calculated, resulting in eight dependent diffusivity parameters: First, the mean (M) of each diffusivity parameter across all voxels within the skeleton was computed, producing four mean parameters (in units and mm^2^/s) for each subject (FA_M_, MD_M_, RD_M_, and AD_M_). In a second step, these voxel-based diffusivity parameters were further computed using histogram analyses according to Baykara and colleagues' approach (33): Accordingly, the peak width of the skeletonized diffusion parameters was determined by computing the difference between the 95th and 5th percentile of each of the skeletonized FA, MD, RD, and AD values (PS in mm^2^/s), resulting in four additional diffusion parameters for each subject (FA_PS_, MD_PS_, RD_PS_, and AD_PS_). For a better comparability to the FA parameters, all mean and PS values of MD, RD, and AD were multiplied with a factor of 1,000. An overview of DTI preprocessing steps and DTI metrics computation is given in Figure 1.

### Statistics

All statistical analyses were performed with SPSS 24. Based on our hypotheses, group differences in FA, MD, RD, and AD parameters (FA_M_, MD_M_, M_RD_, M_AD_, FA_PS_, MD_PS_, RD_PS_, and AD_PS_) and cognitive performance (VLMT_rec, VLMT_con, VLMT_recog, ANT_RTcor, ANT_err, and TMT_RTexe) between patients and controls as well as between IDH-mutated (IDHmut) and wildtype patients (IDHwt) were analyzed using independent-samples *t* test, tested one-sided with a significance level of *p* < 0.05. In addition, standardized effect sizes (ES) with the respective confidence intervals (CIs, Hedges bias corrected) were computed.

The relationship between WM integrity (FA_M_, MD_M_, RD_M_, AD_M_, FA_PS_, MD_PS_, RD_PS_, and AD_PS_) and cognitive performance (VLMT_rec, VLMT_con, VLMT_recog, ANT_RTcor, ANT_err, and TMT_RTexe) was analyzed using Pearson's partial correlation analyses separately for patients and controls, tested one-tailed with a significance level of *p* < 0.05 and controlled for effects of age, education, and tumor volume. Furthermore, intercorrelations between respective diffusivity parameters (FA_M_-FA_PS_, MD_M_-MD_PS_, RD_M_-RD_PS_, and AD_M_-AD_PS_) were computed using partial correlations corrected for age, education and tumor volume.

## Results

### Diffusion Parameters and Cognitive Performance

**Figure 2:**
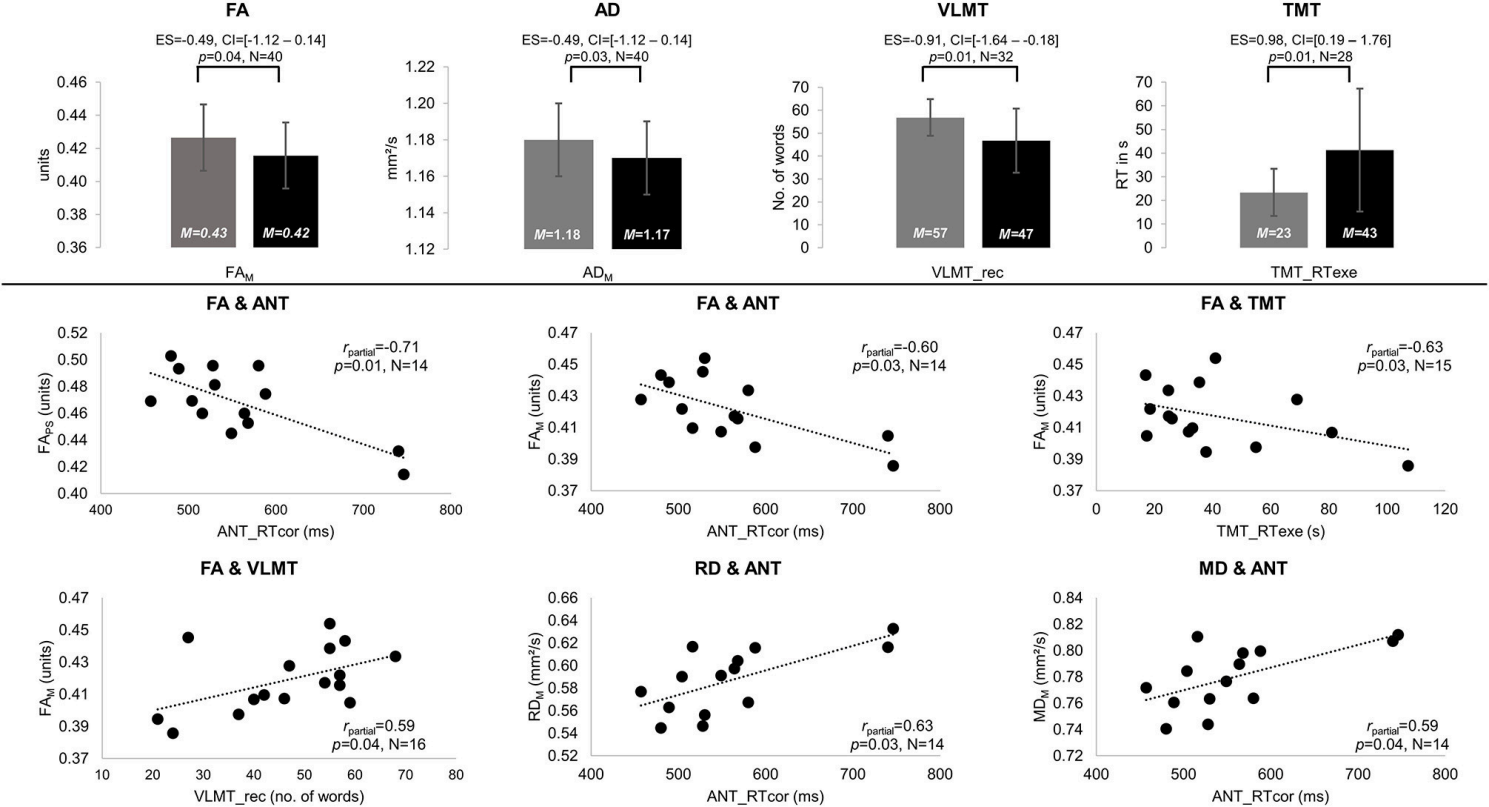
Significant results of between-group and partial correlation analyses. Significant differences in fractional anisotropy (FA) and axial diffusivity (AD) as well as performance on the Verbal Learning and Memory Test (VLMT, VLMT_rec = number of words recalled) and the Trail-Making Test (TMT, TMT_RTexe = reaction times on differences between TMT parts A and B) are displayed in gray for the control group (CG) and black for patients (PAT) in the upper panel. All significant partial correlations (*r*_partial_) between mean (M) and peak width of skeletonized (PS) FA, mean, radial, and axial diffusivity (MD, RD, and AD), and behavioral performance are visualized for patients in the lower panel. Significances for each analysis (*N* = number of subjects included) were computed at *p* < 0.05, including standardized effect sizes (ES) and confidence intervals (CI).

NAWM of patients and controls differed in anisotropy, with significantly lower FA and AD values in patients compared to controls (FA_M:_ *M*_PAT_ = 0.42 and *M*_CG_ = 0.43 units, *t* = 1.85, *p* = 0.04, ES = – = 49, CI = [−1.12–0.14]; AD_M:_ *M*_PAT_ = 1.17 and *M*_CG_ = 1.18 mm^2^/s, *t* = 2.01, *p* = 0.03, ES = – = 49, CI = [−1.12–0.14]). Significant group differences in cognitive performance were found for verbal learning and task switching (VLMT_rec: *M*_PAT_ = 47 and *M*_CG_ = 57 words, *t* = 2.65, *p* = 0.01, ES = – = 91, CI = [−1.64 to −0.18]; TMT_RTexe: *M*_PAT_ = 43 and *M*_CG_ = 23 s, *t* = −2.54, *p* = 0.01, ES = 0.98, CI = [0.19–1.76]) (see Figure 2). For detailed results on diffusivity and behavioral measures (see Table 2).

**Table 2:** Results of group statistics on differences between patients and controls.

**DV**	CG (***N*** = 20)		PAT (***N*** = 20)						IDHmut (***N*** = 12)		IDHwt (***N*** = 7)					
	***M***	***SD***	***M***	***SD***	***t***	p^*^	**ES**	**CI**	***M***	***SD***	***M***	***SD***	***t***	p^*^	**ES**	**CI**
FA_M_	0.43	0.02	0.42	0.02	1.85	**0.04**	**−0.49**	**[−1.12–0.14]**	0.43	0.02	0.40	0.02	−3.45	**0.002**	–**1.43**	**[**–**2.47−0.04]**
FA_PS_	0.48	0.02	0.46	0.02	1.74	0.05	−0.98	[−1.64 to **–**0.32]	0.47	0.02	0.45	0.02	−1.81	0.05	−0.96	[−1.94**–**0.03]
MD_M_	0.79	0.02	0.79	0.03	0.05	0.48	0.00	[−0.62**–**0.62]	0.78	0.02	0.81	0.02	3.28	**0.002**	**1.43**	**[0.40–2.47]**
MD_PS_	0.29	0.05	0.32	0.06	−1.39	0.09	0.53	[−0.10**–**1.16]	0.29	0.05	0.36	0.06	2.54	**0.01**	**1.24**	**[0.23–2.26]**
RD_M_	0.59	0.03	0.60	0.03	−0.69	0.25	0.33	[−0.33**–**0.95]	0.58	0.03	0.62	0.03	3.55	**0.001**	**1.27**	**[0.26–2.29]**
RD_PS_	0.41	0.03	0.42	0.04	−0.95	0.17	0.28	[−0.35**–**0.90]	0.41	0.02	0.45	0.04	2.60	**0.01**	**1.33**	**[0.31–2.36]**
AD_M_	1.18	0.02	1.17	0.02	2.01	**0.03**	–**0.49**	**[**–**1.12–0.14]**	1.16	0.02	1.18	0.02	2.02	**0.03**	**0.96**	**[**–**0.03–1.94]**
AD_PS_	0.75	0.04	0.74	0.05	0.67	0.25	−0.22	[−0.84**–**0.41]	0.72	0.04	0.75	0.06	1.25	0.11	0.60	[−0.35**–**1.55]
VLMT_rec	57	8	47	14	2.65	**0.01**	–**0.91**	**[**–**1.64−0.18]**	51	11	35	16	−2.35	**0.02**	–**1.29**	**[**–**2.52−0.05]**
VLMT_con	1	2	2	2	−1.32	0.10	0.50	[−0.22**–**1.23]	2	1	3	5	0.41	0.35	0.35	[−0.82**–**1.51]
VLMT_recog	14	1	12	2	1.61	0.06	−1.04	[−1.80 to **–**0.28]	13	2	11	2	−2.24	**0.02**	–**1.23**	**[**–**2.48–0.01]**
ANT_RTcor (ms)	505	64	560	87	−1.28	0.11	0.70	[−0.06**–**1.46]	546	75	613	123	1.21	0.13	0.73	[−0.57**–**2.04]
ANT_F	4	3	3	4	0.68	0.25	−0.27	[−1.01**–**0.48]	3	4	2	1	−0.45	0.33	−0.27	[−1.55**–**1.01]
TMT_RTexe (s)	23	10	43	26	−2.54	**0.01**	**0.98**	**[0.19–1.76]**	32	15	55	38	1.65	0.06	0.91	[−0.30**–**2.12]

*DV, dependent variable; CG, control group; PAT, patient group; IDH, isocitrate–dehydrogenase; IDHmut, IDH-mutated glioma; IDHwt, IDH-wildtype glioma; N, number of included subjects; M, mean; SD, standard deviation; t, value of test statistic; p, significance; ES, effect size; CI, confidence interval; FA, fractional anisotropy; MD, mean diffusivity; RD, radial diffusivity; AD, axial diffusivity; PS, peak width difference of skeletonized diffusivity; VLMT, Verbal Learning and Memory Test; ANT, Attention Network Test; TMT, Trail-Making Test; VLMT_rec, recall; VLMT_con, consolidation; VLMT_recog, recognition; ANT_RTcor, reaction time of correct trials; ANT_F, number of errors; TMT_RTexe, difference in reaction time between TMT-A and TMT-B*.

* *Significant results (p <0.05, one-tailed), ES, and CI are printed in bold*.

### Correlations Between Diffusivity Parameters and Cognitive Performance

Intercorrelations between M and PS diffusion parameters revealed significant associations for FA and MD (CG: FA_M_-FA_PS_: *r*_partial_ = 0.77, *p* < 0.001, and MD_M_-MD_PS_: *r*_partial_ = 0.51, *p* = 0.02; PAT: FA_M_-FA_PS_: *r*_partial_ = 0.80, *p* = 0.003 and MD_M_-MD_PS_: *r*_partial_ = 0.78, *p* = 0.004).

In the patient group, significant correlations were found between diffusion parameters and verbal learning, attention, and task switching. Higher FA values were associated with better performances in verbal learning (FA_M_-VLMT_rec: *r*_partial_ = 0.59, *p* = 0.04), attention (FA_M_-ANT_RTcor: *r*_partial_ = –0.60, *p* = 0.03; FA_PS_-ANT_RTcor: *r*_partial_ = –0.71, *p* = 0.01), and task switching (FA_M_-TMT_RTexe: *r*_partial_ = –0.63, *p* = 0.03). In contrast, higher MD and RD correlated significantly with worse attentional performance (MD_M_-ANT_RTcor: *r*_partial_ = 0.59, *p* = 0.04; RD_M_-ANT_RTcor: *r*_partial_ = 0.63, *p* = 0.03) (see Figure 2). In controls, higher MD values correlated significantly with worse attentional performance (MD_PS_-ANT_err: *r*_partial_ = 0.53, *p* = 0.02).

### IDH-Mutation Status

**Figure 3:**
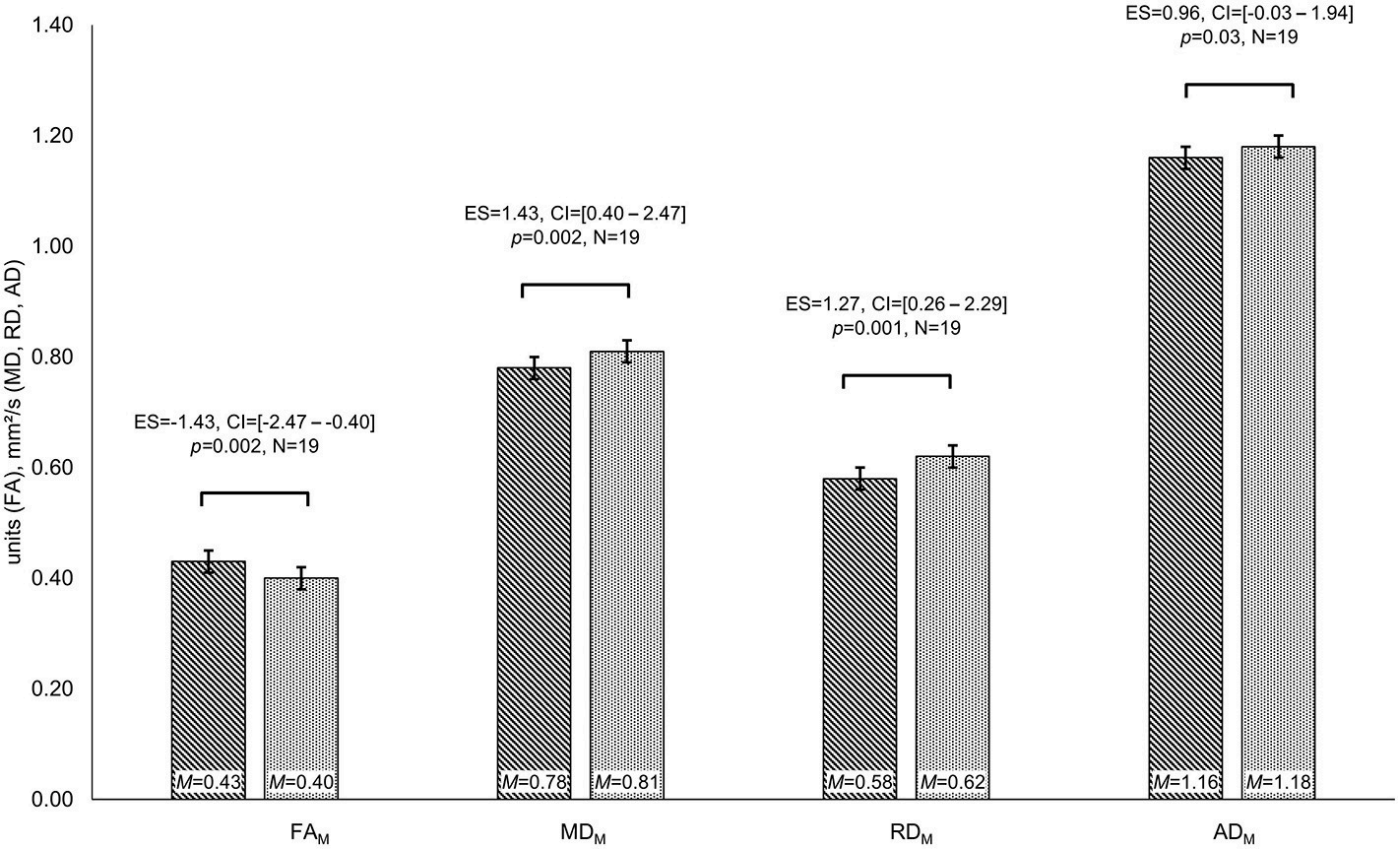
Group differences in diffusivity parameters depending on IDH-mutation status. Significant differences in fractional anisotropy (FA), mean diffusivity (MD), and radial diffusivity (RD) are displayed for patients with isocitrate–dehydrogenase (IDH)-mutation status (lined bar) and IDH-wildtype patients (dotted bar). Significances for each analysis (*N* = number of patients) were computed at *p* < 0.05, including standardized effect sizes (ES) and confidence intervals (CI).

Patients differed in diffusion parameters depending on IDH-mutation status, revealing higher FA (FA_M:_ *M*_IDHmut_ = 0.43 and *M*_IDHwt_ = 0.40 units, *t* = −3.45, *p* = 0.002, ES = −1.43, CI = [−2.47 to −0.04]) as well as lower MD, RD, and AD values for IDH-mutated than for IDH-wildtype gliomas (MD_M:_ *M*_IDHmut_ = 0.78 and *M*_IDHwt_ = 0.81 mm^2^/s, *t* = 3.28, *p* = 0.002, ES = 1.43, CI = [0.40–2.47]; MD_PS:_ *M*_IDHmut_ = 0.29 and *M*_IDHwt_ = 0.36 mm^2^/s, *t* = 2.54, *p* = 0.01, ES = 1.24, CI = [0.23–2.26]; RD_M:_ *M*_IDHmut_ = 0.58 and *M*_IDHwt_ = 0.62 mm^2^/s, *t* = 3.55, *p* = 0.001, ES = 1.27, CI = [0.26–2.29]; RD_PS:_ *M*_IDHmut_ = 0.41 and *M*_IDHwt_ = 0.45 mm^2^/s, *t* = 2.60, *p* = 0.01, ES = 1.33, CI = [0.31–2.36]; AD_M:_ *M*_IDHmut_ = 1.16 and *M*_IDHwt_ = 1.18 mm^2^/s, *t* = 2.02, *p* = 0.03, ES = 0.96, CI = [−0.03–1.94]) (see Figure 3). Furthermore, cognitive performance differed significantly between IDH-mutated and IDH-wildtype patients with regard to verbal recall and verbal recognition (VLMT_rec: *M*_IDHmut_ = 51 and *M*_IDHwt_ = 35 words, *t* = −2.35, *p* = 0.02, ES = −1.29, CI = [−2.52 to −0.05]; VLMT_recog: *M*_IDHmut_ = 13 and *M*_IDHwt_ = 11 words, *t* = −2.24, *p* = 0.02, ES = −1.23, CI = [−2.48–0.01]).

## Discussion

DTI revealed functionally relevant microstructural alterations of NAWM in glioma patients, which agrees with the notion of glioma as a systemic rather than a focal brain disease. Microstructural heterogeneity of NAWM was related to the prognostically relevant IDH-mutation status.

### DTI of NAWM

DTI was used to investigate glioma as systemic brain disease by voxel-wise comparison of extralesional WM of patients and individually matched healthy controls. Prior lesion masking excluded any macroanatomically apparent tumor lesions including perifocal T2- or T1-weighted signal alterations such as caused by edema or perifocal tumor infiltration, which are known to be associated with altered diffusion properties (34, 35).

Agreeing with our hypothesis, diffusion properties of NAWM differed between patients and controls, showing decreases of FA and AD in patients. Furthermore, IDH-mutated gliomas were associated with higher FA, but lower MD, RD, and AD values of NAWM than IDH-wildtype gliomas. These results agree with the notion of a compromised microarchitecture of NAWM in gliomas, with decreases in FA and increases in isotropic diffusion values having been linked to WM degradation (33) and tumor cell infiltration (2, 36). Our results agree with previous findings on decreases of FA and increases of the apparent diffusion coefficient (ADC) at presumed tumor infiltration trajectories along the corticospinal tract in children with diffuse intrinsic pontine glioma (DIPG): Wagner et al. examined patients with DIPG and patients with low-grade brainstem glioma in comparison to controls. They found lower AD and increases in RD at assumed tumor projections remote to the primary lesion site. Decreases of AD were related to axonal damage by disruption of microstructural fiber tract architecture, accompanied by increases of isotropic tissue, which was assumed to reflect diffuse tumor infiltration with increased cellular density in the extracellular matrix (15). Another study applying serial measurements of diffusion properties in children with pontine glioma showed longitudinal changes of diffusion parameters; they suggested those changes to reflect different disease stages, with a transient increase of FA and decrease of ADC relating to transient treatment response, while a subsequent decrease of FA and increase of ADC were regarded to reflect tumor progression (37). Accordingly, Kallenberg et al. described increases in ADC and decreases in FA values in tumor projection areas onto the corpus callosum in glioma patients as an indicator of an occult transcallosal tumor progression (14).

Price and colleagues previously correlated histopathological specimens obtained by image-guided biopsies with DTI metrics. Tumor infiltration was related to increases in the isotropic component, while tumor was associated with reduced anisotropy relative to the contralesional hemisphere. Authors furthermore showed that tumor infiltration occurred in normal-appearing regions on T2-weighted images in 40% of cases (36). Another study described biopsies taken from normal-appearing brain areas with identification of tumor infiltration in half of these specimens (38). Based on those findings, in which histologically proven tumor cell infiltration in NAWM was linked to decreases in anisotropy and increases in diffusivity (MD and RD) values, the diffusion changes found in our study appear to be compatible with previous observations of occult tumor cell infiltration into NAWM, although histopathological evidence cannot be provided here. Although FA is regarded as highly sensitive for microstructural alterations, the scalar measure seems unspecific as to its precise histoanatomical correlate, unless complementary information is available. Interpretation of differences in diffusion values therefore has to be regarded with caution, as values can be ambiguous as they are modulated by multiple factors (39). Moreover, interpretation of diffusion data with regard to tumor pathology is further complicated, as diffusion measures can relate to different pathologies and might offer different possible attributions (39). Related to tumor pathology, diffusion changes might be caused by vasogenic edema, by tumor-infiltration-induced changes of the extracellular milieu with increases in cellular density, or by local tumor effected fiber disruption and secondary Wallerian degeneration. Even neuroplasticity accompanying chronically progressive tumor lesions could be assumed, while aging-related WM changes have to be considered in addition, as well as region-specific dynamics and longitudinal changes during the disease course. Moreover, different tumor-induced processes may even occur in parallel.

However, irrespective of the precise histoanatomical nature of the here observed altered diffusion properties of NAWM, present results indicate that glioma may impact on systemic microstructural WM properties, which cannot be delineated in conventional imaging. Furthermore, cognitive dysfunction was shown not only to depend on local tumor effects but also to be associated with occult WM involvement, which could be regarded as structural correlate of a more widely distributed cognitive network disintegration. It therefore appears promising to monitor and further investigate microstructural characteristics of NAWM in glioma, as it could provide a better understanding of the correlation between the macroscopically apparent tumor lesion and its clinical manifestation and might offer additional non-invasive biomarker *in vivo*. Whole-brain WM characteristics in glioma patients should be further explored with regard to histopathological and molecular genetic specifications, as they might reflect local tumor characteristics which may precede locally apparent disease dynamics.

### Cognitive Performance and Its Association With WM Integrity

In accordance with previous research, treatment-naive patients in our study presented with cognitive impairment in all cognitive domains tested. Most distinctive deficits were found in verbal memory and executive functions, which have been described to manifest early in the disease course, and often prior to other tumor-related symptoms (21, 22). Although it has to be assumed that tumor site had an impact on the type of cognitive domain affected, further subanalysis of different tumor locations and behavioral performance was not attempted, not only in view of the small sample sizes but also because this study did not aim at a clinicotopographical correlation with tumor site. Instead, we intended to investigate the potential impact of a focal tumor lesion on systemic microstructural WM integrity and its relation to cognitive dysfunction. Systemic WM disintegrity contributing to cognitive decline is known from normal aging (40), WM diseases, or neurodegenerative disorders (33, 41). In view of spatiotemporally widely distributed cognitive network representations (42), we thus aimed to investigate cognitive deficits as functional surrogates of tumor-induced systemic network alterations based on occult WM involvement.

Agreeing with this notion, decreases in FA and increases in MD and RD as potential signs of WM disintegrity were associated with worse cognitive performance in our study. Previous studies suggested cognitive performance in glioma patients to be regarded as a prognostically relevant factor, with better performances on verbal memory and executive tests correlating with longer survival rates and less aggressive tumor growth in high-grade gliomas (43, 44). Moreover, the post-treatment cognitive status has been suggested as a clinical predictor of tumor recurrence, even in the absences of structural evidence in conventional imaging (45). While subtle cognitive impairment is often neglected in clinical routine, standardized neuropsychological assessment combined with information about microstructural NAWM characteristics may improve treatment monitoring by increasing the clinical sensitivity to disease dynamics.

### IDH-Mutation Status

With IDH-mutation having emerged as a major prognostic disease marker (18, 19, 46), the more favorable prognosis in IDH-mutated gliomas has been attributed to slower local tumor growth rates (46), to anatomical predelection sites that are more accessible to extensive tumor resection (47, 48), or to a less infiltrative nature of diffuse tumor cell migration (17). In our sample, IDH-wildtype patients showed lower FA, but higher MD, RD, and AD values of NAWM as compared to IDH-mutated glioma, which may indicate a less preserved microstructural integrity of NAWM than in IDH-mutated patients: With glioma cells invading the intercellular space along WM fibers (4), initial fiber displacement is ensued by axonal damage and disruption of the blood–brain barrier and leads to vasogenic edema. While increases in isotropic diffusion values have previously been linked to tumor cell infiltration with increased cellular density in the extracellular matrix and later on with vasogenic edema, decreases in anisotropy components have been related to increased fiber density or axonal damage and fiber disruption (14, 15, 37, 38). Higher MD, RD, and AD but lower FA values in our IDH-wildtype glioma patients thus might reflect an increased cellular density in the extracellular matrix due to tumor cell invasion with fiber compression and ensuing axonal damage. Decreased AD values in IDH-mutated glioma might relate to the slower and presumably less invasive growth behavior, more prone to remote axonal degeneration due to secondary Wallerian degeneration. Microstructural disintegrity and occult tumor cell invasion cannot be proven based on the present data. However, our results agree with findings by Price and colleagues (17), who found differences in peritumoral isotropic and anisotropic diffusion properties related to IDH-mutation status, suggesting IDH-mutated glioblastomas to have a less invasive phenotype than IDH-wildtype glioblastomas (17). With the IDH1 R132 (Arginine 132) mutation leading to an accumulation of 2 hydroxyglutarate, magnetic resonance spectroscopy (MRS) allows the measurement of this oncometabolite *in vivo* (49, 50), which has been applied for non-invasively predicting IDH-mutation status in glioma patients (51) and for investigating remote glioma cell infiltration *in vivo* (52–54). Recent studies even used MRS to identify tumor-specific metabolic profiles and investigated metabolic–transcriptional alterations under consideration of genetic profiles (55–57). A multimodal approach combining MRS and a diffusion-based characterization of NAWM microarchitecture may allow future studies to further specify histopathological correlates of NAWM diffusion properties *in vivo*.

Microstructural alterations of NAWM were accompanied by worse verbal memory performance in IDH wildtype as compared to IDH-mutated patients, which supports previous observations of higher cognitive dysfunction related to tumor grade (21, 23) and is in line with increasing evidence of the prognostic predominance of molecular rather than histopathological tumor characteristics (18, 46).

### Limitations

It cannot be excluded that pharmacological effects caused by corticosteroids or anticonvulsants may have influenced behavioral performance and diffusion values in the patient group. NAWM integrity was however associated with cognitive performance even in subjects without medication. Furthermore, a recent study examined patients with gliomas and found increased neurocognitive dysfunction associated with grade IV as compared to grade II or III tumors, which was found to be independent of steroids and anti-epileptic medication (23). In addition, dexamethasone has been reported to affect diffusion only within tumor, but not in normal brain, and steroid-related reductions of MD have been observed in peritumoral tissue in high-grade gliomas, whereas FA values have remained unchanged despite corticosteroid treatment (58). It may thus be assumed that decreases in MD under steroid treatment are more likely related to reductions in vasogenic edema, whereas anisotropy values appear to be less sensitive to steroid effects and to reflect structural fiber integrity independent thereof.

A further limitation is the small sample size and tumor heterogeneity in our cohort, which has impeded further analyses of the potential impact of different tumor characteristics such as tumor grade or histoanatomical specifications on structural and functional properties of NAWM. Nevertheless, a particular strength of the present study is the matching of patients with healthy subjects controlling for age, gender, lesion volumes, and educational level. A further appeal of the method used is the potential clinical perspective, as the ROI-independent approach reduces interobserver variability and allows integration of patients with different tumor locations and sizes. Even among the small number of subjects examined in this study, differences in behavioral performance as well as in diffusion properties of NAWM were observed. While the strength of the effect sizes further reinforce the informative value of the present results, larger patient studies are needed to validate and further extend the present findings.

## Conclusions

DTI revealed microstructural alterations of NAWM in glioma patients in association with cognitive dysfunction, adding to local tumor effects. Microstructural heterogeneity of NAWM was furthermore associated with IDH-mutation status, which might reflect a more preserved microstructural integrity of NAWM and less occult systemic tumor burden in IDH-mutated as compared to IDH-wildtype glioma. Diffusion-based phenotyping microstructural properties of NAWM may aid in estimating occult WM involvement and in disease monitoring and should be considered as a complementary biomarker in glioma.

## Data Availability

The datasets for this manuscript are not publicly available because the raw MRI data contain non-anonymized, subject-specific personal information. Converted and anonymized data can be provided upon request. Requests to access the datasets should be directed to Kerstin Jütten, kjuetten@ukaachen.de.

## Ethics Statement

The study was carried out in accordance with the standards of Good Clinical Practice with written informed consent from all subjects. All subjects gave written informed consent in accordance with the Declaration of Helsinki. The protocol was approved by the local ethics Committee of the Medical Faculty of the University of the RWTH Aachen (EK 294/15).

## Author Contributions

CH-N, VM, HC, and SG contributed to the conception and design of the study. Data collection and evaluation were carried out by KJ and VM (neuropsychological assessment) as well as by MW, KJ, CH-N, HP, and FB (MRI data). Statistical analyses and visualization were performed by KJ. The manuscript was written by CH-N, KJ, and VM. All authors critically reviewed and approved the manuscript.

### Conflict of Interest Statement

The authors declare that the research was conducted in the absence of any commercial or financial relationships that could be construed as a potential conflict of interest.
